# Passaging of gingival fibroblasts from periodontally healthy and diseased sites upregulates osteogenesis-related genes

**DOI:** 10.1007/s13577-023-00995-3

**Published:** 2023-10-26

**Authors:** Gerasimos D. Karlis, Ton Schoenmaker, Nektarios Tsoromokos, Olaf E. Veth, Bruno G. Loos, Teun J. de Vries

**Affiliations:** 1 Department of Periodontology, Academic Centre for Dentistry Amsterdam (ACTA), University of Amsterdam and Vrije Universiteit, Gustav Mahlerlaan 3004, 1081 LA Amsterdam, The Netherlands; 2 Private Practice for Periodontology and Implantology, Zwolle, The Netherlands

**Keywords:** Cell passaging, Gingival fibroblasts, Periodontitis

## Abstract

To investigate biological processes of the periodontium, in vitro primary cell models have been established. To study the biology of the gingiva, primary gingival fibroblast cell models are widely used. For such experiments, cells need to be expanded and passaged. A key assumption is that primary cells maintain most of their original characteristics they have in situ. The aim of this research is to explore the impact of early passaging on selected gene expression of human gingival fibroblast cells. For this purpose, gene expression from the outgrowth of the resected tissues until the fourth passage was followed for nine tissue samples, from both healthy and diseased sites. Micrographs were taken from the cultures, RNA was extracted from the samples of each passage and quantitative PCR was performed for selected genes representing various biological processes. Epithelial cells were present during the first outgrowth, but were no longer present in the second passage. Our results indicate that the morphology of the gingival fibroblast cells does not change with passaging and that passages 2–4 contain only gingival fibroblasts. Gene expression of *M-CSF*, *TNF-α*, *TLR4*, *POSTN* and *FAPα* was unchanged by passaging, the expression of *IL-6*, *IL-1β* and *TLR2* decreased due to passaging and the expression of in particular the selected osteogenesis genes (*ALP*, *RUNX2*, *Osteonectin*, *COL1A*), *OPG* and *MKI67* increased with passaging. Worldwide, use of the same passage in laboratory experiments using primary cell cultures is the standard. Our results support this, since for certain genes, in particular osteogenesis genes, expression may alter solely due to passaging.

**Supplementary Information:**

The online version contains supplementary material available at 10.1007/s13577-023-00995-3.

## Introduction

Periodontitis is a plaque-related inflammatory disease of the tooth-supporting structures, which affects about 42% of the dentate of US adults aged 30–79 years in a moderate form [1] and 11.2% of the global population in a severe form [2]. The main characteristic of periodontitis is bone loss due to an enduring inflammation, which can ultimately lead to tooth loss [3–5].

Periodontitis starts as a disbalance between the host immune response and the bacterial load. The initially protective inflammatory response toward the bacteria can become a chronic, non-resolving inflammation [6–8]. This non-resolving inflammation further activates several inflammatory cascades, which result in production of many, potentially damaging, cytokines, such as interleukin-1β (IL-1β), tumor necrosis factor-alpha (TNF-α), interleukin-6 (IL-6), and others [9]. The expression of these pro-inflammatory cytokines and mediators during inflammation influences the interplay between osteogenesis and osteoclastogenesis; pro-inflammatory cytokines such as IL-1β and TNF-α increase the production of receptor activator of nuclear factor kappa-Β ligand (RANKL), which regulates the osteoclast differentiation and function via binding on its receptor RANK [10]. Next, such inflammatory cytokines can also induce osteoclast formation in a direct manner [11, 12]. An increase of the ratio of RANKL to osteoprotegerin (OPG), a cytokine that binds to RANKL to prevent the activation of the RANK, contributes to the bone loss that is seen in periodontitis [13, 14]. In a healthy situation, osteoclastogenesis is counterbalanced by osteogenesis. Following bone resorption, osteoblasts are recruited to the site, where they secrete and mineralize new matrix. Osteoblasts are derived from undifferentiated mesenchymal cells. The protein runt-related transcription factor 2 (RUNX2) serves as an early osteogenesis marker and is a key transcription factor associated with osteoblast maturation [15]. The production of the enzyme alkaline phosphatase (ALP), a protein required for mineral deposition, is initiated by the mature osteoprogenitor cells and is therefore used as an intermediate osteogenesis marker [16]. Once the mature osteoblasts are active, they secrete a matrix containing collagen type 1 and several growth factors and continue with the deposition of osteoid [16]. Osteonectin is a late osteogenesis marker, initiating mineralization and promoting mineral crystal formation [17]. During the inflammatory condition of periodontitis, the ratio of osteoclastogenesis to osteogenesis is disturbed and leans toward the former.

To explore these intricate biological interactions, elegant in vitro models have been established [18, 19]. Relevant cell systems have been used to investigate the interplay between osteogenesis, osteoclastogenesis, and resident cells of the periodontium, such as gingival fibroblasts [20, 21], periodontal ligament fibroblasts [22, 23], and alveolar bone cells [24–26]. For these assays, primary cells of passage 3–7 are typically used. Biologically relevant parameters that have been studied are the responses of gingival fibroblasts to bacteria or bacterial products [18–21, 23] and their capacity to contribute to osteogenesis [20, 26, 27] and osteoclastogenesis [20, 22, 26].

Due to the limited amount of periodontal tissues that can be obtained, cells are often expanded after the initial outgrowth over several passages to obtain a sufficient cell number [27]. The impact of the number of times that primary cells have been passaged can be complex and most likely dependent on multiple factors, such as the source of primary cells, the culture conditions, and the intended use of the cells [28]. Eventually, biological processes such as telomere shortening and senescence will determine how long primary cells can be passaged. For tumor cells, specialized in escaping telomere shortening and senescence, it is more common to be cultured for 25–30 passages or more [29]. Rheumatoid arthritis synovial fibroblasts on the contrary, when cultured for five to six passages, showed significantly increased and reduced gene expression of 7–10% of the genes when compared with passage 1 [30].

To the best of our knowledge, the effects of passaging have hardly been addressed in the dental literature. One research group [31] studied the gene expression and quantified the cytokines of healthy gingival fibroblasts from passages 1 to 10. However, their findings were based on primary cell cultures from one donor.

The current study aims to explore the impact of passaging on the gene expression of healthy and diseased derived human gingival fibroblasts. Our null hypothesis is that there will be no significant differences in gene expression levels between passages 1 and 4 of these gingival fibroblasts.

## Materials and methods

The study was approved by the medical–ethical board of the Academic Center for Dentistry Amsterdam (ACTA) (protocol number: 2020305) and performed in accordance with the ethical standards as laid down in the 1964 Declaration of Helsinki and its later amendments.

### Subjects

Gingival fibroblasts (GF) were obtained from seven individuals (Table 1) (age 38–68 years) who underwent periodontal surgery or tooth extraction in a referral private practice for periodontology and implantology between February and November 2021. The subjects were categorized as either periodontally diseased or periodontally healthy. The periodontally diseased subjects were diagnosed with severe periodontitis (grade III according to the latest classification [3]. All subjects were systemically healthy, were non-smokers or quit at least 1 year before the collection of the tissues, were not pregnant, and did not receive systemic antibiotics for at least the last 6 months. Informed consent was obtained from all individuals.

**Table 1:** Subjects’ characteristics

	Age (years)	Sex (female/male)	Probing pocket depth (mm)	Bleeding on probing (yes/no)
Sample 1, subject 1	64	Female	12	Yes
Sample 2, subject 2	68	Male	7	Yes
Sample 3, subject 3	51	Female	7	Yes
Sample 4, subject 4	61	Female	11	Yes
Sample 5, subject 2	68	Male	3	No
Sample 6, subject 4	61	Female	3	No
Sample 7, subject 5	45	Female	3	No
Sample 8, subject 6	38	Male	2	No
Sample 9, subject 7	56	Male	3	No

Samples 1– 4 are categorized as diseased and samples 5–9 as healthy

### Gingival tissue areas and tissue sample collection

There were three types of collected gingival tissues: (i) diseased sites with probing pocket depth (PPD) ≥ 6 mm, with bleeding on probing (BOP) collected from a periodontitis patient; (ii) healthy sites with PPD ≤ 4 mm, without BOP from a periodontitis patient; and (iii) healthy sites with ≤ 4 mm, without BOP from periodontally healthy subjects.

Periodontal surgery was performed in periodontitis patients with residual pockets ≥ 6 mm with BOP, at least 2 months after non-surgical periodontal treatment. Paramarginal/intrasulcular incisions were performed and the interproximal gingival papillae that adhered to the root surface were dissected. Dissected tissues corresponding to the deepest site were collected and transferred to a plastic sterile 50 mL tube containing culture medium (Dulbecco’s modified Eagle medium (DMEM, Gibco BRL, Paisley, Scotland), supplemented with 10% Fetal Clone I serum (HyClone, Logan, UT, USA) and 2% antibiotics: 100 U/mL penicillin, 100 μg/mL streptomycin, and 250 ng/mL amphotericin B (Sigma, St. Louis, MO, USA)). When the periodontal flap was extended to an adjacent tooth with PD ≤ 4 mm without BOP to accommodate accessibility, visibility, and flexibility of the flap, a non-inflamed interproximal tissue specimen was obtained (healthy site from periodontitis patient).

Non-inflamed specimens (PD ≤ 4 mm without BOP) from periodontally healthy subjects were collected during (i) extraction of teeth with peri-apical complications, (ii) extraction of non-restorable teeth, or (iii) clinical crown lengthening procedures. Attention was paid to collecting the same gingival structure as during the periodontal surgery. The collected tissues were clinical waste material.

### Tissue cultures

The tubes with the specimens were transferred to the laboratory within 12 h, cut into small pieces with a sterile scalpel knife in a flow cabinet, and divided into two wells in a six-well dish with 2 mL culture medium. The six-well dishes were stored in a humidified atmosphere of 5% CO_2_ in air at 37° C. The wells were refreshed every 3–4 days. When the cultures were confluent (after 3 weeks), the medium was removed, the cells were washed with PBS, trypsinized with 0.5 mL trypsin (Gibco) at 37° C, and monitored until cells detached. 1/5^th^ of the cells were stored at − 80° C (RNA of passage 1). The rest of the cells were transferred to a 75 cm^2^ flask and stored in a humidified atmosphere of 5% CO_2_ in air at 37° C. The cultures were refreshed every 3–4 days and when the cultures were confluent (after 1–2 weeks), the same protocol was followed for the 75 cm^2^ flask (with the difference that 1% antibiotics were used). This protocol was repeated to obtain cells and RNA samples of passage 3 and 4.

### Real-time quantitative PCR (QPCR)

RNA was extracted from samples using a commercial spin-column kit (RNeasy Mini kit, Qiagen, Düsseldorf, Germany) according to the manufacturer’s protocol. RNA concentration was measured with Synergy HT spectrophotometer (BioTek Instruments Inc., Winooski, VT, USA). One hundred nanograms of RNA was used in the reverse transcriptase reaction which was performed according to the manufacturer’s instructions of the MBI Fermentas cDNA synthesis kit (Vilnius, Lithuania), using both the Oligo(dT)18 and the D(N)6 primers. The Primer Express software, version 2.0 (Applied Biosystems, Foster City, CA, USA), was used to design the real-time PCR primers.

Real-time PCR was performed on the ABI PRISM 7000 (Applied Biosystems). The reactions were performed with 5 ng cDNA in a total volume of 25 mL containing SYBR Green PCR Master Mix, consisting of SYBR Green I Dye, AmpliTaq Gold DNA polymerase, dNTPs with dUTP instead of dTTP, passive reference and buffer (Applied Biosystems), and 300 nM of each primer. After an initial activation step of the AmpliTaq Gold DNA polymerase for 10 min at 94° C, 40 cycles were run of a two-step PCR consisting of a denaturation step at 94° C for 30 s and annealing and extension step at 60° C for 1 min. Subsequently, the PCR products were subjected to melting curve analysis to test if any unspecific PCR products were generated. The PCR reactions of the different amplicons had equal efficiencies. β2-Microglobulin was used as the housekeeping gene. Expression of this gene was not affected by the experimental conditions. Samples were normalized for the expression of β2-microglobulin by calculating the ΔCt, (Ct_gene of interest_ -Ct_β2-microglubulin_) and the expression of the different genes (Table 2) is expressed as the mean relative fold expression 2^−(ΔCt)^.

**Table 2:** Primer sequences used for QPCR experiments

Gene	Primer sequence		Ensembl gene ID^a^
*KRT14*	Forward	5′ TGCCGAGGAATGGTTCTTCACC 3′	ENSG00000186847
	Reverse	5′ GCAGCTCAATCTCCAGGTTCTG 3′	
*KRT5*	Forward	5′ GCTGCCTACATGAACAAGGTGG 3′	ENSG00000186081
	Reverse	5′ ATGGAGAGGACCACTGAGGTGT 3′	
*IL-6*	Forward	5′ CACAATCTGCAGTACCTGCAAGGAT 3′	ENSG00000136244
	Reverse	5′ CCCATAGTGGAAGCGCAGATA 3′	
*IL-1β*	Forward	5′ CATGCGAGCCATCATCGA 3′	ENSG00000206439
	Reverse	5′ CATGCGAGCCATCATCGA 3′	
*TLR2*	Forward	5′ GGCTTCTCTGTCTTGTGACCG 3′	ENSG00000137462
	Reverse	5′ GAGCCCTGAGGGAATGGAG 3′	
*TLR4*	Forward	5′ CTGCAATGGATCAAGGAACCAG 3′	ENSG00000136869
	Reverse	5′ CCATTCGTTCAACTTCCACCA 3′	
*TNF-α*	Forward	5′ CCCAGGGACCTCTCTCTAATCA 3′	ENSG00000232810
	Reverse	5′ GCTTGAGGGTTTGCTACAACATG 3′	
*M-CSF*	Forward	5′ CCGAGGAGGTGTCGGAGTAC 3′	ENSG00000184371
	Reverse	5′ AATTTGGCACGAGGTCTCCAT 3′	
*RANKL*	Forward	5′ CATCCCATCTGGTTCCCATAA 3′	ENSG00000120659
	Reverse	5′ GCCCAACCCCGATCATG 3′	
*OPG*	Forward	5′ CTGCGCGCTCGTGTTTC 3′	ENSG00000164761
	Reverse	5′ ACAGCTGATGAGAGGTTTCTTCGT 3′	
*RUNX2*	Forward	5′ ATGCTTCATCGCCTCAC 3′	ENSG00000124813
	Reverse	5′ ACTGCTTGCAGCCTTAAAT 3′	
*ALP*	Forward	5′ GCTTCAAACCGAGATACAAGCA 3′	ENSG00000162551
	Reverse	5′ GCTCGAAGAGACCCAATAGGTAGT 3′	
*Osteonectin*	Forward	5′ TACATCGGGCCTTGCAAATAC 3′	ENST00000231061
	Reverse	5′ AGGGTGACCAGGACGTTCTTG 3′	
*COL1A*	Forward	5′ TCCAACGAGATCGAGATCC 3′	ENSG00000108821
	Reverse	5′ AAGCCGAATTCCTGGTCT 3′	
*MKI67*	Forward	5′ GAAAGAGTGGCAACCTGCCTTC 3′	ENSG00000148773
	Reverse	5′ GCACCAAGTTTTACTACATCTGCC 3′	
*POSTN*	Forward	5′ CCCAGCAGTTTTGCCCATT 3′	ENSG00000133110
	Reverse	5′ TGTGGTGGCTCCCACGAT 3′	
*FAPα*	Forward	5′ AGCGACTACGCCAAGTACTATGC 3′	ENSG00000078098
	Reverse	5′ CATCATGAAGGGTGGAAATGG 3′	
*β2-Microglobulin*	Forward	5′ CGGGCATTCCTGAAGCTGA 3′	ENSG00000106927
	Reverse	3′ GGATGGATGAAACCCAGACACATAG 3′	

### Statistics

GraphPad Prism software (version 9.0.2, La Jolla, CA, USA) was used to analyze the data sets. Means and standard error of the means (SEM) were calculated and used for the presentation of the data in the figures. The normality of data distribution was assessed with the D’Agostino–Pearson test. All the data were analyzed with repeated measures one-way ANOVA, followed by Tukey’s multiple comparison test. Tests were performed over the four passages. Differences were considered significant at *p* < 0.05.

## Results

### Patient population

Age and sex variables of the study are outlined in Table 1. There were three groups. Group 1 included diseased sites from a periodontitis patient (samples 1–4), group 2 included healthy sites from a periodontitis patient (samples 5–7), and group 3 included healthy sites from a subject without periodontitis (samples 8 and 9). For all genes assessed, there seemed to be no differences between samples from groups 2 and 3 (*n* = 2 and *n* = 3 respectively), albeit that this could not be statistically tested due to the too low *n* (Suppl. Figures 1 and 2). Based on this, we decided to merge the data into one group of healthy samples. Samples 2 and 5 and samples 4 and 6 were sampled from the same patient (matched samples). The age of the patients varied between 38 and 68 years. The PPD of the diseased samples varied between 7 and 12 mm and of the healthy samples between 2 and 3 mm. All the diseased sites showed BOP and none of the healthy sites showed BOP. The majority of the subjects in the diseased group were female and in the healthy group male.

### Gingival fibroblast cultures

**Fig. 1:**
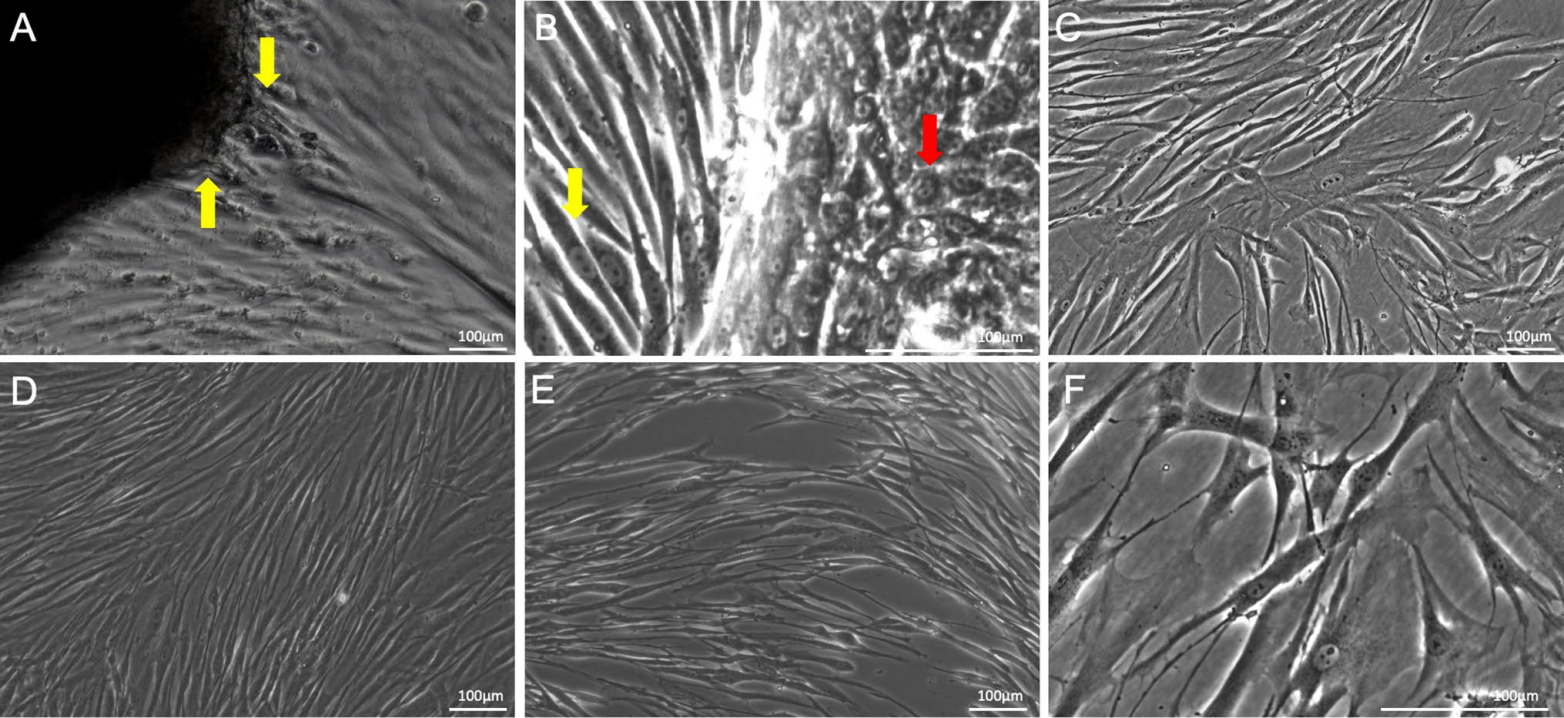
Morphological characteristics of gingival fibroblasts for passages 1–4. **A** Outgrowth of gingival cells with fibroblast morphology from tissue fragments (yellow arrows). **B** At passage 1, elongated cells, typical for gingival fibroblasts cells (yellow arrows), as well as cuboidal cells, typical for epithelial cells (red arrows), were observed. **C** At passage 2, **D** passage 3, and **E** passage 4, no epithelial cells were observed, but only elongated gingival fibroblast cells. The shape and size of the cells did not differ in different passages. **F** Gingival fibroblasts of passage 2 at higher magnification. The scale bar represents 100 μm at the micrograph **A**–**F**. Micrographs are representative for nine different GF sources

Within 7–10 days of incubation of the tissues, solitary GF started to appear, likely detaching from the tissue fragments or grown out of these. Elongated cells with fibroblast morphology were observed deriving from the tissue fragments (Fig. 1A; yellow arrows). Within 3 weeks, the cultures were 80% confluent and ready for passaging. Besides, in these cultures, a population of epithelial cells was regularly observed, but not in all cases (Fig. 1B; red arrows). After the first trypsinization, only gingival fibroblast-like cells and no epithelial cells were observed anymore in the cultures (Fig. 1C, D, E, and F). The cultures were observed under a light microscope and photographs were taken (Fig. 1). The size and shape of the gingival fibroblasts did not differ from passage 1 to passage 4.

### Decreased *IL-6*,* IL-1β*, and Toll-like receptor 2 (*TLR2)* expression and increased *OPG* expression with passaging

**Fig. 2:**
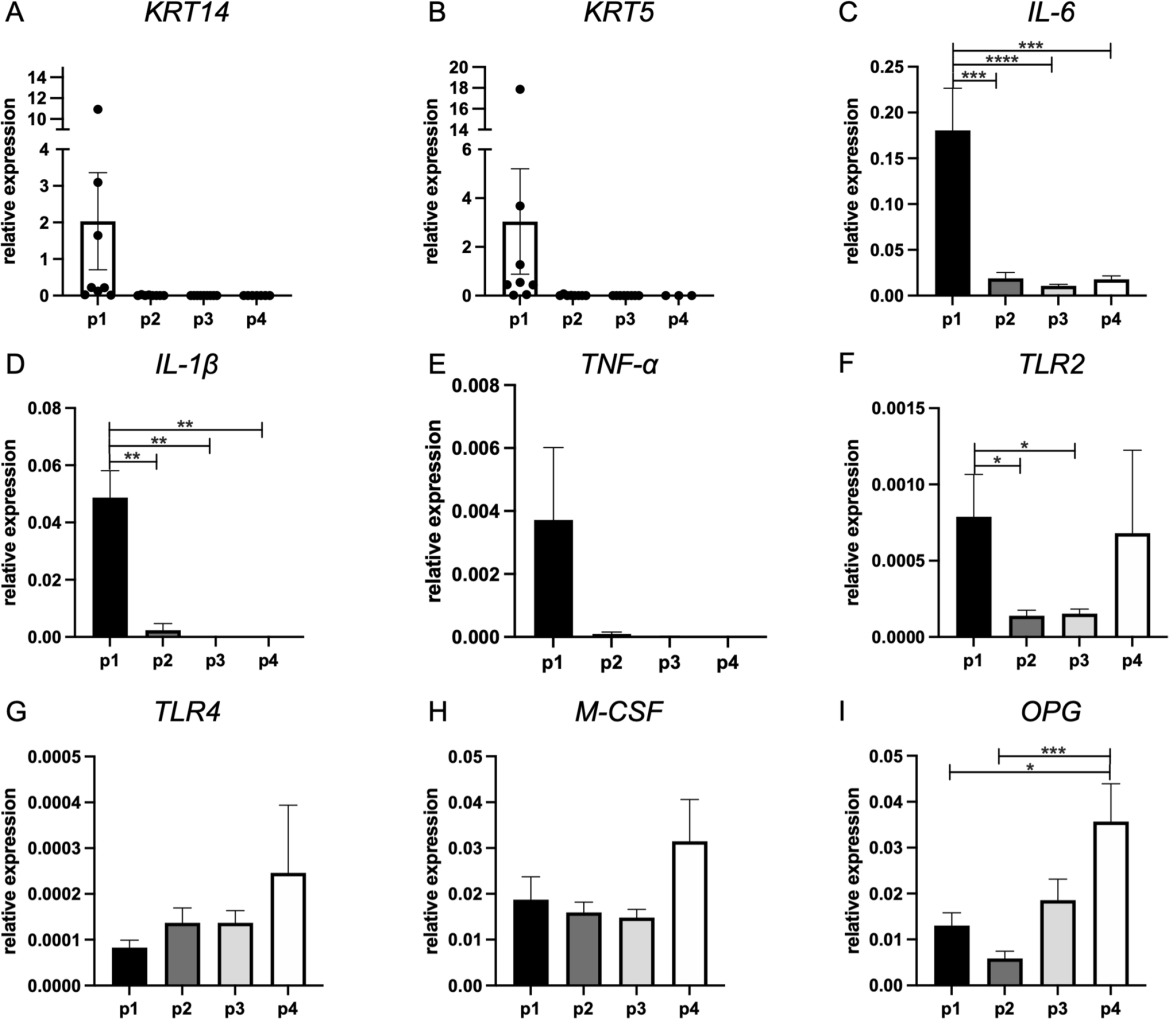
Passaging decreases the expression of some inflammation-related genes. Gene expression of **A** *KRT14*, **B** *KRT5*, **C** *IL-6*, **D** *IL-1β*, **E** *TNF-α*, **F** *TLR2*, **G** *TLR4*, **H** *M-CSF*, and **I** *OPG*. *n* = 9 (diseased and healthy sites combined). Significant results are shown (black bars). *p < 0.05, **p < 0.01, ***p < 0.005, ****p < 0.001. For *KRT14* and *KRT5* gene expression, individual biological samples are shown, since not all first outgrowths contained keratinocytes

**Fig. 3:**
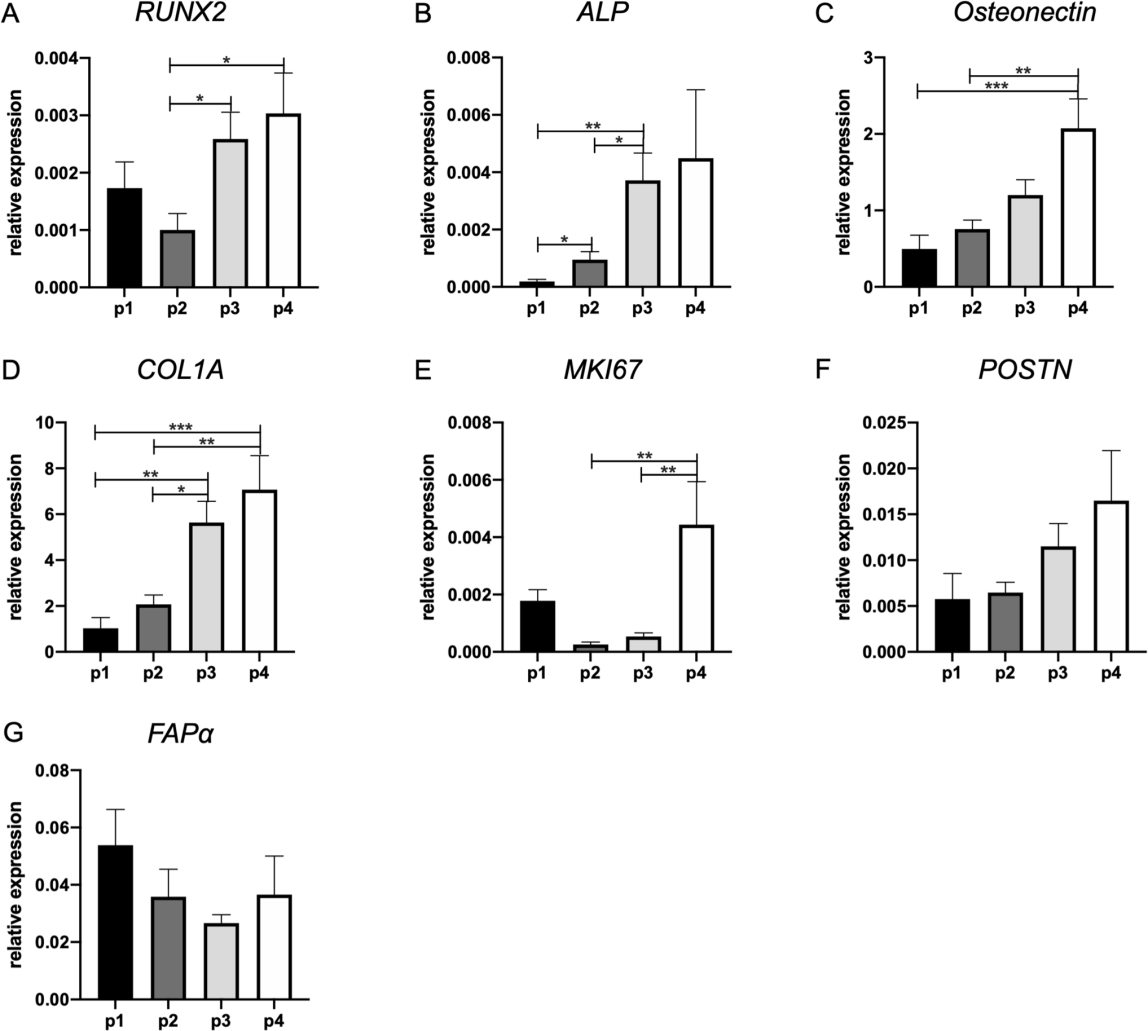
Osteogenesis-related genes consistently increase their expression in higher passages. Gene expression of **A** *RUNX2*, **B** *ALP*, **C** osteonectin, **D** *COL1A*, **E** cell cycle marker *MKI67*, mesenchymal cell markers **F** *POSTN*, and **G** *FAPα*. *n* = 9 (diseased and healthy sites combined). Significant results are shown (black bars). *p < 0.05, **p < 0.01, ***p < 0.005

The RNA that was isolated from all the samples from passages 1 to 4 was used to measure the gene expression of the genes of interest with qPCR (Table 2). These genes are representative markers for biological processes such as cell’s origin (*keratin 14 (KRT14)*, *keratin 5 (KRT5)*, *fibroblast activation protein-α* (*FAPα*), *periostin (POSTN)*), extracellular matrix and bone formation (*collagen I alpha (COL1A)*, *RUNX2*, *ALP*, and osteonectin), inflammation (*IL-1β*, *TNF-α*, *IL-6*, *TLR2*, *Toll-like receptor 4 (TLR4)*), and cytokines for homing and differentiation of osteoclast precursor cells (*macrophage colony-stimulating factor (M-CSF)*, *OPG*, *RANKL*). In Figs. 2 and 3, the expression of the genes and the differential changes related to the passaging are presented, including all the samples (*n* = 9).

In passage 1, *keratin 14* and *keratin 5* were highly expressed in three samples, moderately expressed in another three, and extremely lowly expressed in the last three samples (Fig. 2A, B). From passages 2 to 4, *KRT14* and *KRT5* were not detectable, a finding that corresponds with the light microscopy results.

*IL-6* and *IL-1β* were highly detectable in passage 1, significantly reduced in passage 2, and remained low in passages 3 and 4 (Fig. 2C and D). The same trend was observed for *TNF-α* (Fig. 2E). *Toll-like receptor 2* (*TLR2*) expression significantly decreased from passage 1 to passage 2 (Fig. 2F) and in passage 3 remained at low levels of detection. In passage 4, the gene expression was again comparable to passage 1. *Toll-like receptor 4* (*TLR4*) (Fig. 2G) and *M-CSF* (Fig. 2H) gene expression showed no differences with passaging. *OPG*, an osteoclastogenesis inhibitor factor that prevents the activation of RANK–RANKL was significantly increased in passage 4 compared to passages 1 and 2 (Fig. 2I). Expression of *RANKL* was also measured but its detection was very late, needing > 37 cycles with qPCR, a range where results are inaccurate. In some samples, *RANKL* was not expressed. Given the limited expression of *RANKL*, it is not presented in the figures. Analysis of the gene expression of the gingival fibroblasts derived from diseased and healthy sites as separate groups showed the same tendency in the results (Suppl. Figures 3, 4, 5, and 6). *IL-6* expression was analyzed and compared between healthy and diseased gingival fibroblasts per passage and found not to differ (Suppl. Figure 7).

### Gene expression of the extracellular matrix and osteogenic markers increases at higher passages

The expression of extracellular matrix and osteogenic genes was also measured (Fig. 3). The early osteogenic marker *RUNX2* was significantly increased at passages 3 and 4 in comparison to passage 2 (Fig. 3A). The expression of *ALP*, an intermediate osteogenic factor of osteogenesis, was also increased from passage 1 to passage 2 and elevated even further from passage 2 to passage 3 (Fig. 3B). The late osteogenic factor *osteonectin* was also elevated at passage 4 in comparison with passages 1 and 2 (Fig. 3C). Furthermore, *COL1A*, a gene that encodes the major component of type I collagen, a fibrillar collagen found in most connective tissues, was elevated from passage 1 to passage 3 and even more at passage 4 (Fig. 3D).

### Proliferation marker gene *MKI67* increases at higher passages

*MKI67* expression was assessed as a marker gene for proliferation (Fig. 3E). This gene was found to be more highly expressed in passage 4 compared to passages 2 and 3.

### Mesenchymal markers *POSTN* and *FAPα* are expressed in gingival fibroblasts cultures, but not affected by passaging

Mesenchymal markers *POSTN* (Fig. 3F) and *FAPα* (Fig. 3G) genes were found to be expressed in all passages. Expression of *POSTN* and *FAPα* was stable with passaging.

### Healthy vs. diseased

As an extra analysis, we studied gene expression by dividing the nine samples into two groups, samples from sites with active periodontitis vs samples from healthy sites. The same analysis of the epithelial and fibroblast lineage markers (Figs. 2 and 3) was now subdivided into healthy (Suppl. Figures 3 and 5) and diseased (Suppl. Figures 4 and 6) samples. The results (Suppl. Figures 3, 4, 5, and 6) were in the same line as the previous analysis of all the samples together (Figs. 2 and 3). *IL-6* expression was separately analyzed and compared between healthy and diseased gingival fibroblasts per passage and found not to differ (Suppl. Figure 7).

## Discussion

In this study, we explored the effect of cell expansion and subsequent passaging on the gene expression of human-derived gingival fibroblasts from gingival samples. The rationale of the study was to investigate if early passages following initial outgrowth changes the biological properties of cells that are used in biological assays. With the present results, making use of a limited number of preselected genes, we can distinguish three patterns. First of all, some genes are not affected by passaging, such as the lineage genes *FAPα* and *POSTN*, but also *TNF-α*, *TLR4*, and *M-CSF*. The second group contains genes that are downregulated during passaging. These include *IL-6, IL-1β*, and *TLR2*. Thirdly, a group of genes including *OPG* and *MKI67*, the genes associated with osteogenesis, *RUNX2*, *ALP*, *COL1A*, and o*steonectin*, are upregulated during passaging. Notably, our samples were a mixed population during the first outgrowth, with mainly gingival fibroblasts and some gingival epithelial cells. By visual inspection, but also confirmed by the absence of typical keratocyte markers *KRT14* and *KRT5*, samples of passages 2–4 contained only cells with a fibroblast morphology. Given the low number of cells in early passages,we were not able to confirm whether the fibroblast-like morphology resembles one particular cell type. For cells from the human palate, it has been shown by single cell sequencing that different fibroblast cell populations exist [32]. Such novel technology holds promise for future fine-detailing of cultured fibroblasts. Based on our findings, we reject our hypothesis that passaging would not affect the gene expression of the gingival cells. Early effects, the changes that are found primarily in the first passage, could be attributed to the presence of epithelial cells.

**Fig. 4:**
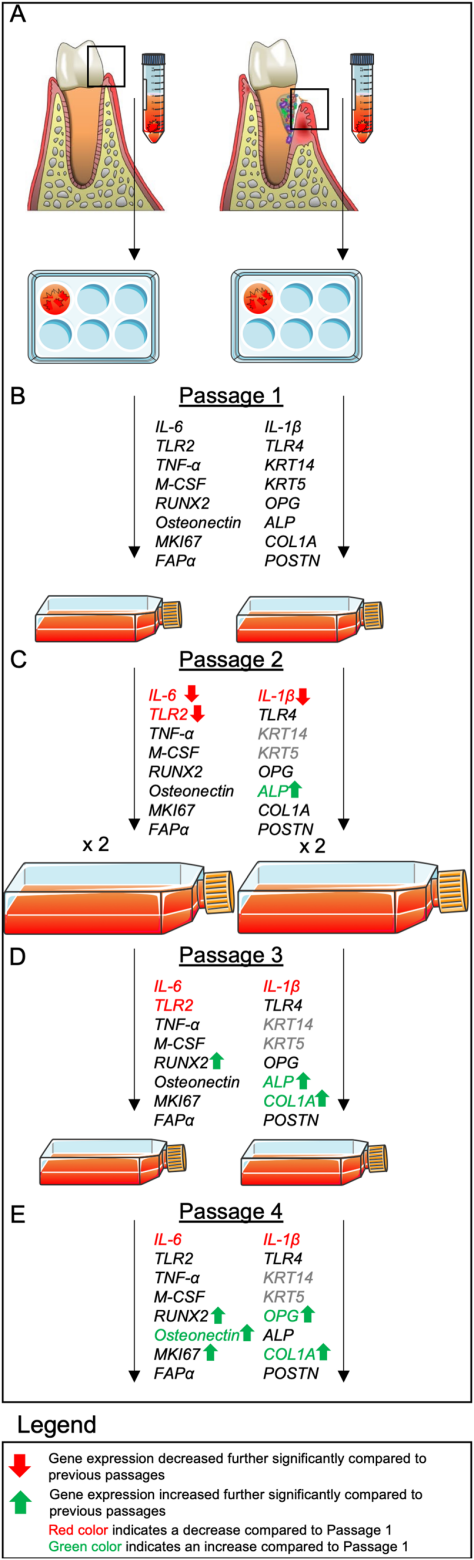
Passaging of gingival fibroblast cells decreases the expression of *IL-6*, *IL-1β*, and *TLR2* and increases the expression of all osteogenesis-related genes. **A** Sample collection. **B** Passage 1. **C** Passage 2. **D** Passage 3. **E** Passage 4. Red color indicates a decrease and green an increase compared to Passage 1. Gray color indicates that the gene is not expressed anymore. Arrows indicate a further increase/decrease compared to previous passages

In our study, we used gingival fibroblasts originating from periodontally diseased sites (samples 1–4) and healthy sites (samples 5–9). The samples from healthy sites include gingival tissues from periodontitis patients and healthy subjects. Sub-analysis of the two different groups of healthy samples did not show any difference in gene expression, so they were grouped. We analyzed the results in two separate groups (diseased and healthy, Suppl. Figures 3, 4, 5, 6, and 7) and as one group (*n* = 9, Figs. 2 and 4). In both analyses, we concluded that inflammatory and pathogen recognition markers (*IL-6, IL-1β* and *TLR2*) decrease and osteogenesis markers (*RUNX2*, *ALP*, Osteonectin, and *COL1A*) increase their expression with passaging. Therefore, the changes in gene expression seem to correlate to passaging rather than to the disease status of the tissue. Our results are in line with a previously published case report [31] where gene expression of pro-inflammatory cytokines such as *IL-1α*, *IL-1β*, *IL-6*, *IL-8*, and *TNF-α* from healthy gingival fibroblasts from one donor was studied in relation to passaging. These genes were analyzed both at mRNA and protein levels. They found that *IL-6* was consistently expressed in all passages (passages 1 to 10) but its cytokine production decreased with passaging, with the largest decrease from passage 1 to passage 2. In the current study, we confirm and strengthen this single observation of one donor with primary cells from nine different samples, originating from seven patients. A possible explanation of the sharp drop in *IL-6 and Il-1β* expression could be due to the presence of epithelial cells. However, the presence of epithelial cells in the samples does not correlate with a significantly higher *IL-6* or *IL-1β* expression*.* We showed additionally that the effect of passaging is independent of the periodontal condition of the gingival tissues (Suppl. Figures 3, 4, 5, 6, and 7) and that the expression of the osteogenic markers is also affected (Fig. 3). It could be plausible to expect that the gene expression levels of *IL-6* would be lower in healthy gingival fibroblasts compared to the diseased ones. Although the current study was not designed to explore this aspect, we found that *IL-6* gene expression does not differ between gingival fibroblast cultures from healthy and diseased sites per passage, and that passaging leads to the same trends in *IL-6* expression in both groups (healthy versus diseased) (Suppl. Figure 7). A previous study [33] also observed that diseased and healthy periodontal tissues, from a group of periodontitis patients compared to periodontally healthy subjects, had similar levels of *IL-6* transcription and protein. However, in this study the gingival tissues were used as they were sampled and not homogenous for a specific cell type.

Although we showed that passaging affects the gene expression of some inflammation-related genes and all of the osteogenesis-related genes that were included, it is beyond the scope of this article to investigate whether these differences in gene expression affect biological assays such as osteogenesis and osteoclastogenesis assays. In fact, this is not possible to test at least not for the first passages, given the restricted number of cells of these first passages. We showed also that cultures from passage 2 to passage 4 contained only cells with fibroblast morphology, but whether they are homogenous in cell types cannot be concluded and should be assessed in future work using single cell sequencing [32].

A limitation of the current study is that we cultured the cells for a limited number of passages, up to passage 4. Therefore, assays with a biologically relevant read-out, such as osteoblastic differentiation, ELISAs of cytokines, and osteoclast differentiation when using co-cultures with human monocytes could not be used. This is an inevitable shortcoming of this study that focuses only on mRNA expression. Many researchers use primary gingival fibroblast cells from passage 1 to 4 [34–37]; however, some research groups use cells from later passages [38, 39]. A study [40] that compared the protein expression of gingival fibroblasts, such as IL-6, IL-8, TNF-α and MMP-3, from passage 4 to 5 (early passage cells) compared to passage 30–35 (late passage cells) found no significant differences. On the other hand, an advantage of the current study is that we measured the gene expression of the outgrowth of the tissues.

The effect of passaging seems to have a gradual effect on most of the tested genes. Concerning the inflammation-related genes, the differences between passages were very limited, with the exception of *IL-6* and *IL-1β*. Here, stable expression after passage 1 was observed. The other genes assessed hardly differed. On the other hand, the effect on the expression of the osteogenesis-related genes seems to be more prominent. Based on our results, experiments with primary cells to study biological processes should be encouraged but well controlled. In future studies, we plan to explore the role of gingival fibroblast cells derived from healthy and diseased sites in terms of osteogenesis and osteoclastogenesis potential.

## Conclusion

Our results suggest that passaging of gingival fibroblast cells derived from healthy and diseased sites has some effects on the expression of inflammation, pathogen recognition, and osteogenesis-related genes (Fig. 4). Most of the differences appear from the first passage, for instance the decreased expression of the *IL-6* and *IL-1β*. On the contrary, the expression of *OPG*, *RUNX2*, *ALP*, osteonectin, and *COL1A* was increased gradually with passaging. Although not possible to assess biological parameters in the present study, despite the expression differences seen here, passages 3 and 4 derived cells, such as those used by many research groups worldwide, could still be seen as a good model to study biological assays that resemble the fibroblast function of the gingiva, such as extracellular matrix production, response to bacteria [18, 19, 23], osteogenesis, and osteoclastogenesis in disease models[20]. We also confirmed that passages 2–4 only contained outgrowth of fibroblast-like cells. Given the fact that some of the gene expressions responded to passaging, we recommend to standardize the passage number in all experiments using biological replicates of various donors.

### Supplementary Information

Below is the link to the electronic supplementary material.

**Supplementary file 1: Figure S1. **Gene expression of inflammation-related genes of gingival fibroblasts deriving from periodontally healthy gingiva from non-periodontitis patients (H) and periodontally healthy gingiva from periodontitis patients (HP). Gene expression of (**A**) *IL-6*, (**B**) *IL-1β*, (**C**) *TNF-α*, (**D**) *TLR2*, (**E**) *TLR4*, (**F**) *M-CSF* and (**G**) *OPG*. *n*=2 and *n*=3 of H and HP respectively.

**Supplementary file 2: Figure S2. **Gene expression of osteogenesis-related genes of gingival fibroblasts deriving from periodontally healthy gingiva from non-periodontitis patients (**H**) and periodontally healthy gingiva from periodontitis patients (HP). Gene expression of (**A**) *RUNX2*, (B) *ALP*, (**C**) Osteonectin, (**D**) *COL1A*, (**E**) *MKI67*, (**F**) *POSTN*, and (**G**) *FAPα.* *n*=2 and *n*=3 of H and HP respectively.

**Supplementary file 3: Figure S3. **Passaging decreases gene expression of *IL-6* in gingival fibroblasts deriving from healthy gingiva. Gene expression of (A) *KRT14*, (**B**) *KRT5*, (**C**) *IL-6*, (**D**) *IL-1β*, (**E**) *TNF-α*, (**F**) *TLR2*, (**G**) *TLR4*, (**H**) *M-CSF* and (**I**) *OPG*. n=5 (healthy sites from periodontitis patients and healthy subjects). Significant results are shown (black bars). **p < 0.01.

**Supplementary file 4: Figure S4. **Gene expression of inflammation-related genes of gingival fibroblasts deriving from periodontally diseased tissues. Gene expression of (**A**) *KRT14*, (**B**) *KRT5*, (**C**) *IL-6*, (**D**) *IL-1β*, (**E**) *TNF-α*, (**F**) *TLR2*, (**G**) *TLR4*, (**H**) *M-CSF* and (**I**) *OPG*. *n*=4.

**Supplementary file 5: Figure S5. **Expression of osteogenesis-related genes of gingival fibroblasts deriving from healthy sites is increased with passaging. Gene expression of (**A**) *RUNX2*, (**B**) *ALP*, (**C**) Osteonectin, (**D**) *COL1A*, (**E**) *MKI67*, (**F**) *POSTN*, and (**G**) *FAPα*. *n*=5 (healthy sites from periodontitis patients and healthy subjects). Significant results are shown (black bars). *p < 0.05

**Supplementary file 6: Figure S6. **Passaging increases the expression of the osteogenesis-related genes of gingival fibroblast cells deriving from diseased sites. Gene expression of (**A**) *RUNX2*, (**B**) *ALP*, (**C**) Osteonectin, (**D**) *COL1A* and (**E**) *MKI67*, (**F**) *POSTN*, and (**G**) *FAPα*. *n*=4. Significant results are shown (black bars). *p < 0.05

**Supplementary file 7: Figure S7. **Gene expression of *IL-6* does not differ between gingival fibroblasts deriving from healthy and diseased sites. Gene expression of *IL-6*. n=5 for healthy and *n*=4 for diseased gingiva. Significant results are shown (black bars). **p < 0.01

## Data Availability

The raw data supporting the conclusions of this article will be made available by the authors, without undue reservation.
