# Supplementary figures and images for: Passaging of gingival fibroblasts from periodontally healthy and diseased sites upregulates osteogenesis-related genes
Source: Hum Cell. 2023 Oct 26;37(1):193–203. doi: 10.1007/s13577-023-00995-3 (PMC10764533; doi:10.1007/s13577-023-00995-3)

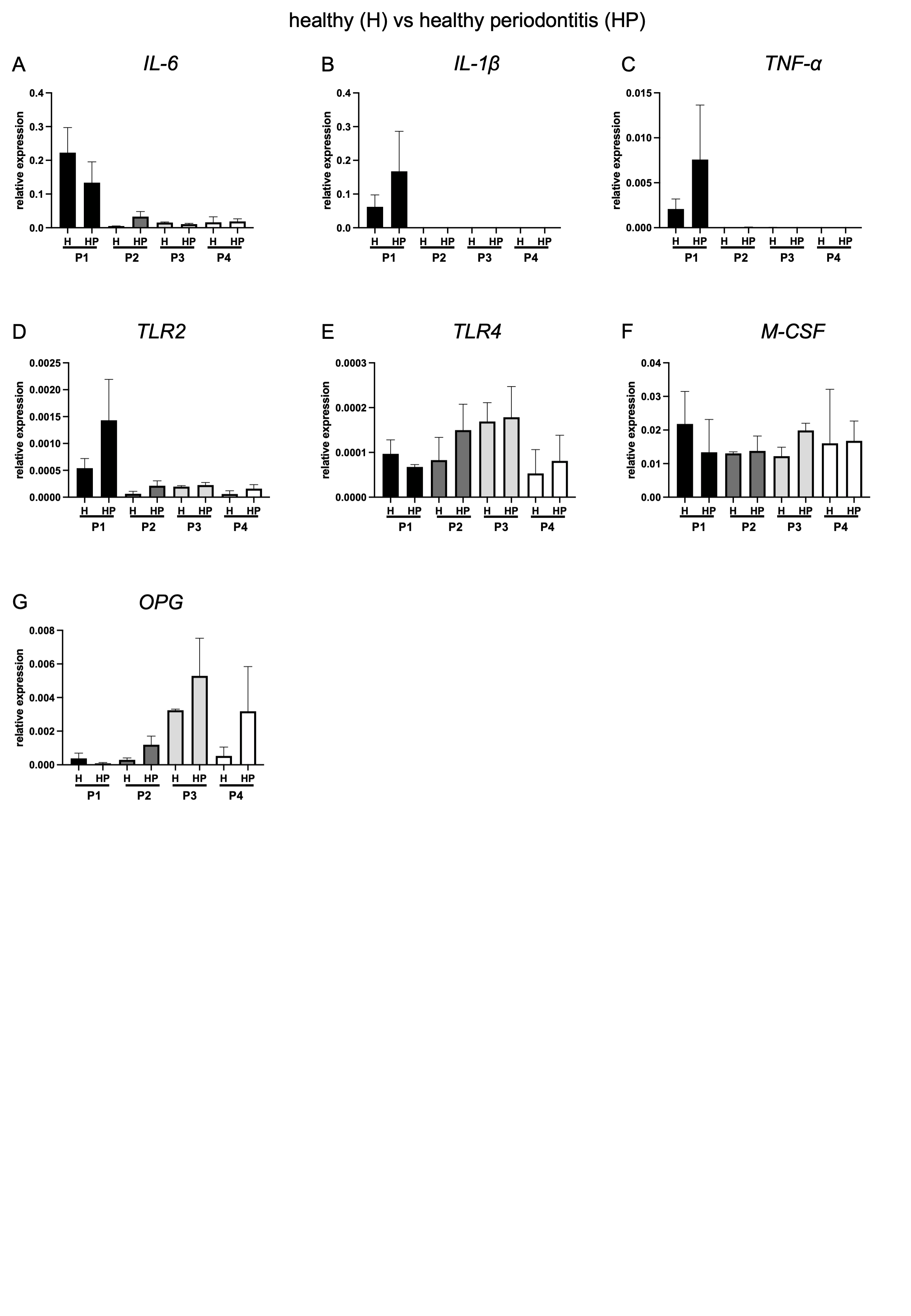

Supplement: Supplementary file 1 — Figure S1. Gene expression of inflammation-related genes of gingival fibroblasts deriving from periodontally healthy gingiva from non-periodontitis patients (H) and periodontally healthy gingiva from periodontitis patients (HP). Gene expression of (A) IL-6, (B) IL-1β, (C) TNF-α, (D) TLR2, (E) TLR4, (F) M-CSF and (G) OPG. n=2 and n=3 of H and HP respectively. [file 13577_2023_995_MOESM1_ESM.tiff]

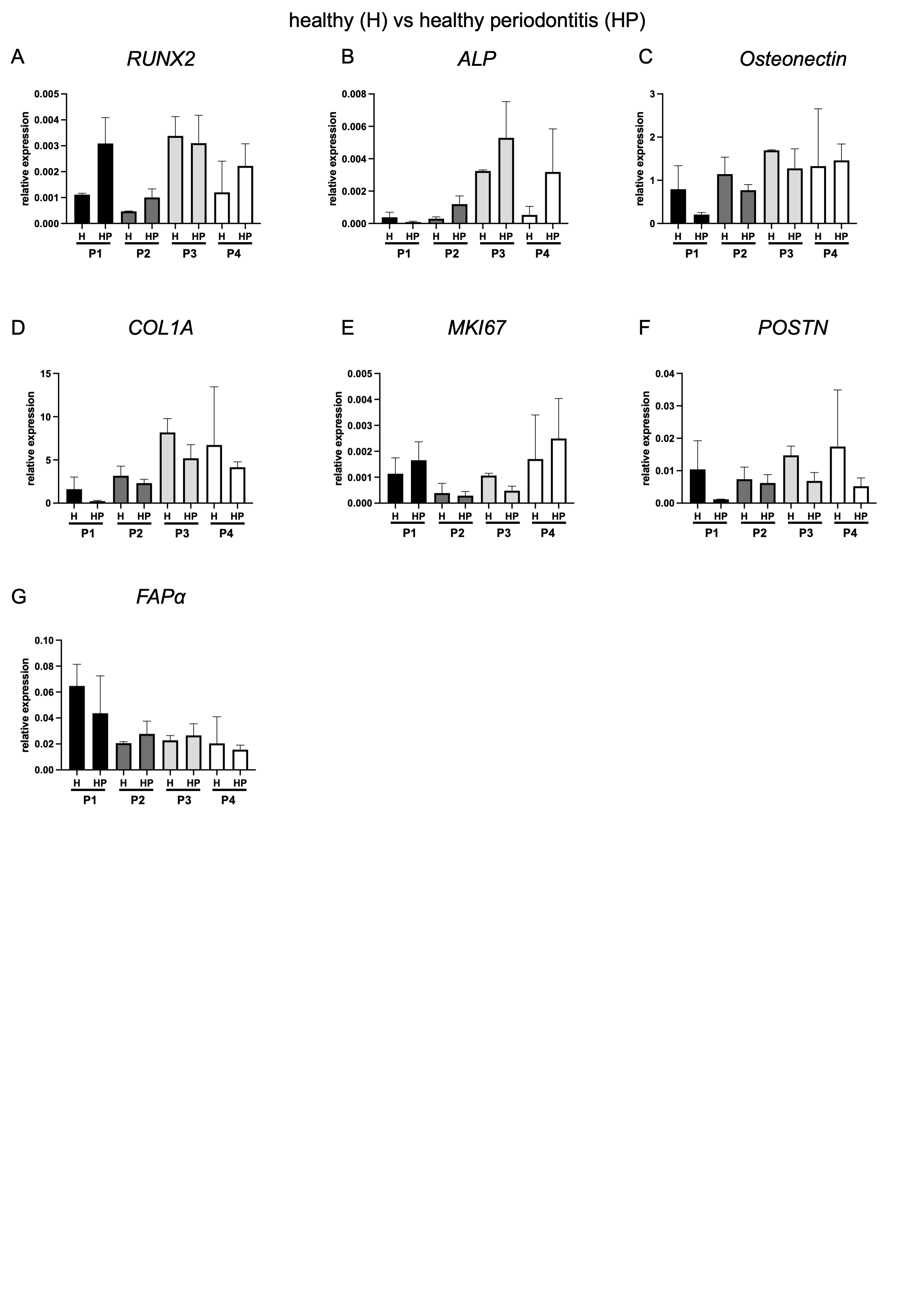

Supplement: Supplementary file 2 — Figure S2. Gene expression of osteogenesis-related genes of gingival fibroblasts deriving from periodontally healthy gingiva from non-periodontitis patients (H) and periodontally healthy gingiva from periodontitis patients (HP). Gene expression of (A) RUNX2, (B) ALP, (C) Osteonectin, (D) COL1A, (E) MKI67, (F) POSTN, and (G) FAPα. n=2 and n=3 of H and HP respectively. [file 13577_2023_995_MOESM2_ESM.tiff]

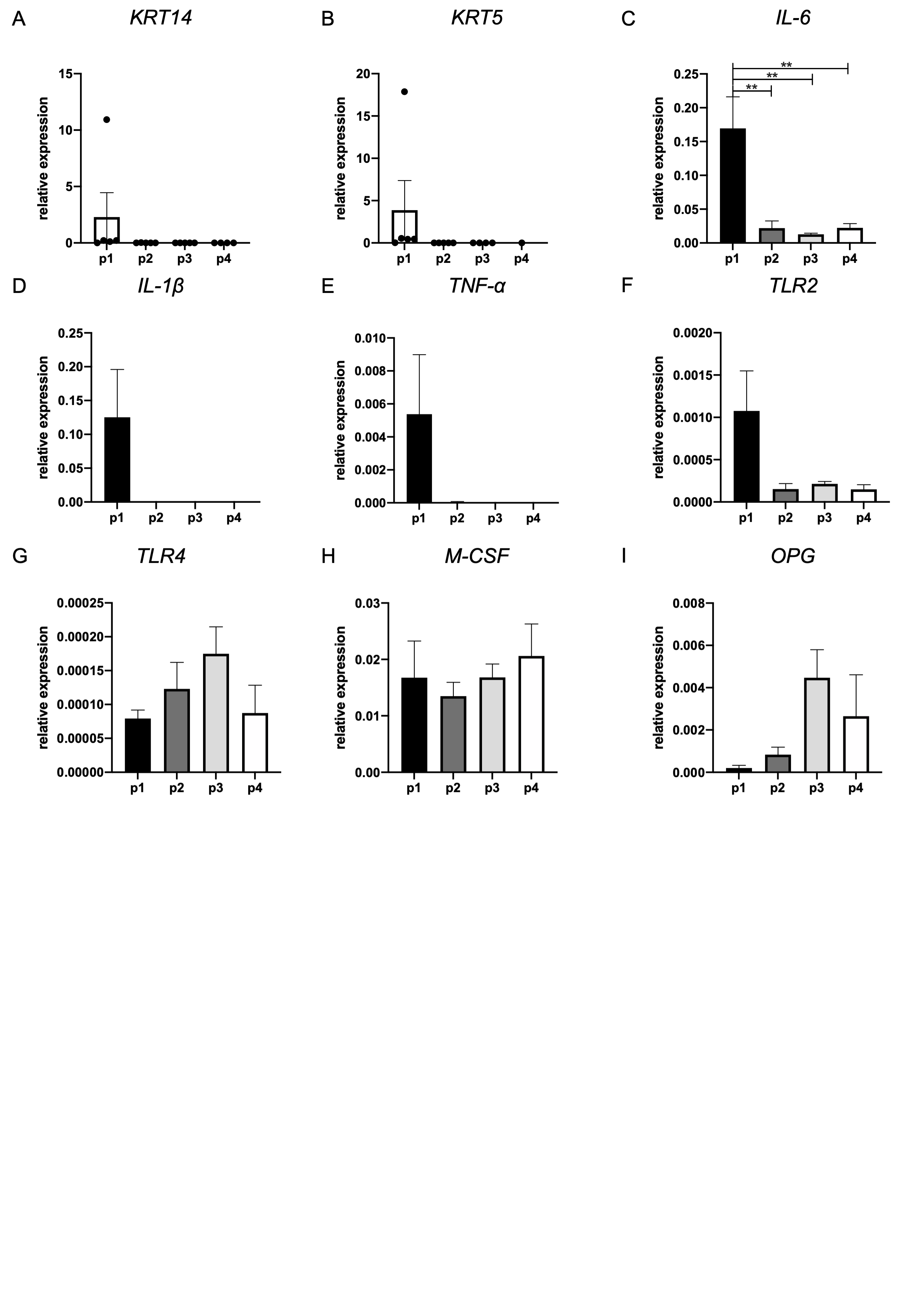

Supplement: Supplementary file 3 — Figure S3. Passaging decreases gene expression of IL-6 in gingival fibroblasts deriving from healthy gingiva. Gene expression of (A) KRT14, (B) KRT5, (C) IL-6, (D) IL-1β, (E) TNF-α, (F) TLR2, (G) TLR4, (H) M-CSF and (I) OPG. n=5 (healthy sites from periodontitis patients and healthy subjects). Significant results are shown (black bars). **p < 0.01. [file 13577_2023_995_MOESM3_ESM.tiff]

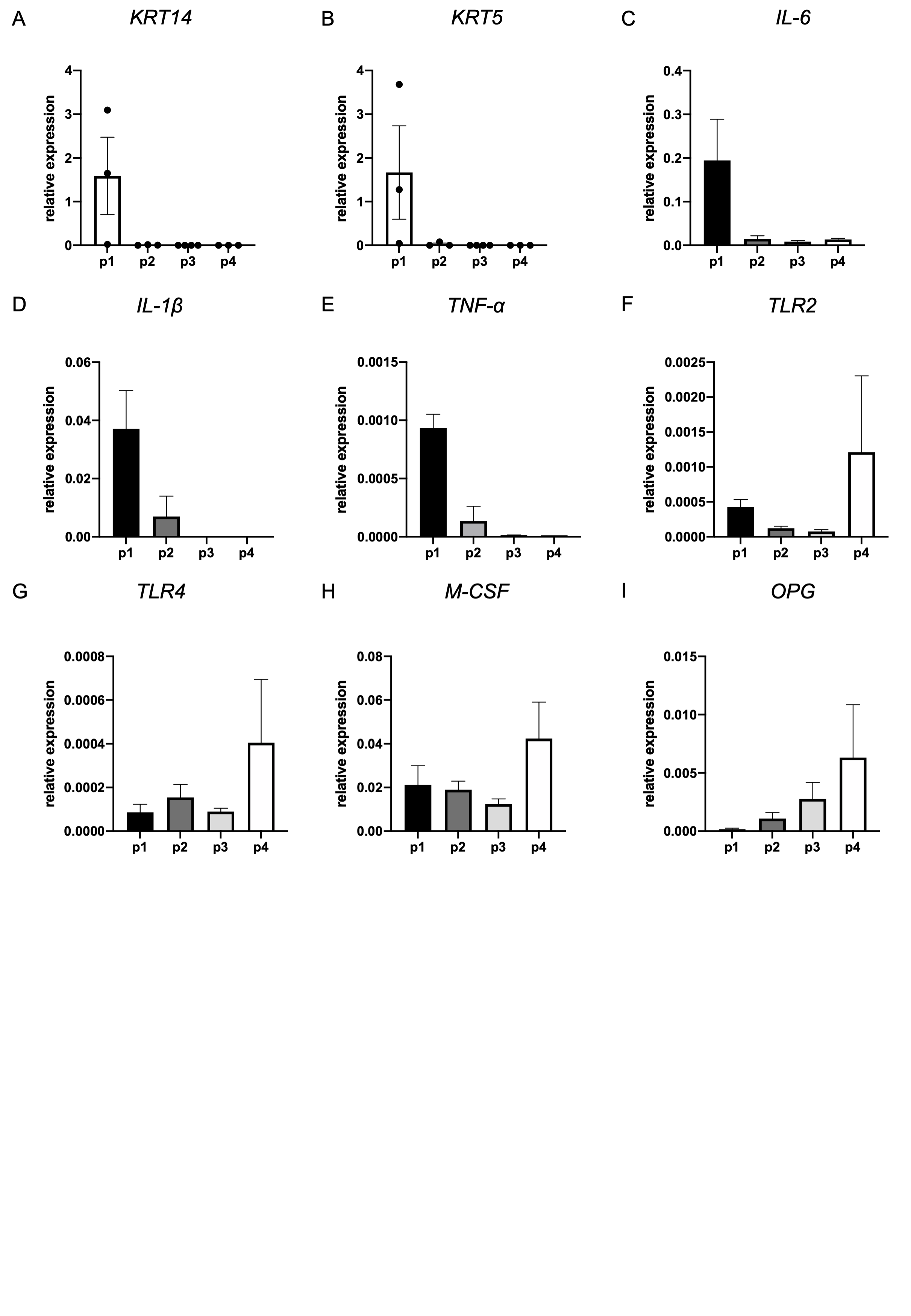

Supplement: Supplementary file 4 — Figure S4. Gene expression of inflammation-related genes of gingival fibroblasts deriving from periodontally diseased tissues. Gene expression of (A) KRT14, (B) KRT5, (C) IL-6, (D) IL-1β, (E) TNF-α, (F) TLR2, (G) TLR4, (H) M-CSF and (I) OPG. n=4. [file 13577_2023_995_MOESM4_ESM.tiff]

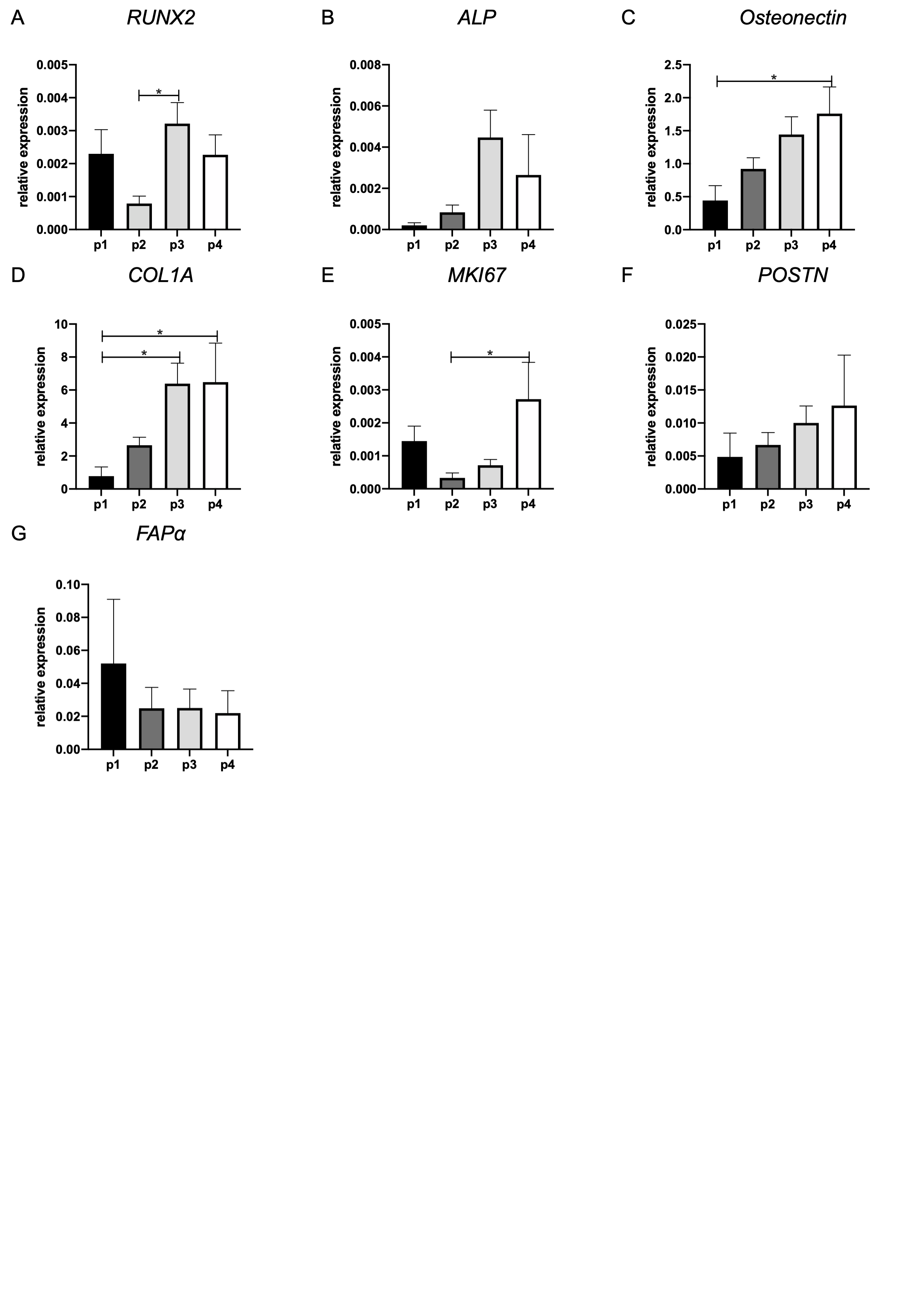

Supplement: Supplementary file 5 — Figure S5. Expression of osteogenesis-related genes of gingival fibroblasts deriving from healthy sites is increased with passaging. Gene expression of (A) RUNX2, (B) ALP, (C) Osteonectin, (D) COL1A, (E) MKI67, (F) POSTN, and (G) FAPα. n=5 (healthy sites from periodontitis patients and healthy subjects). Significant results are shown (black bars). *p < 0.05 [file 13577_2023_995_MOESM5_ESM.tiff]

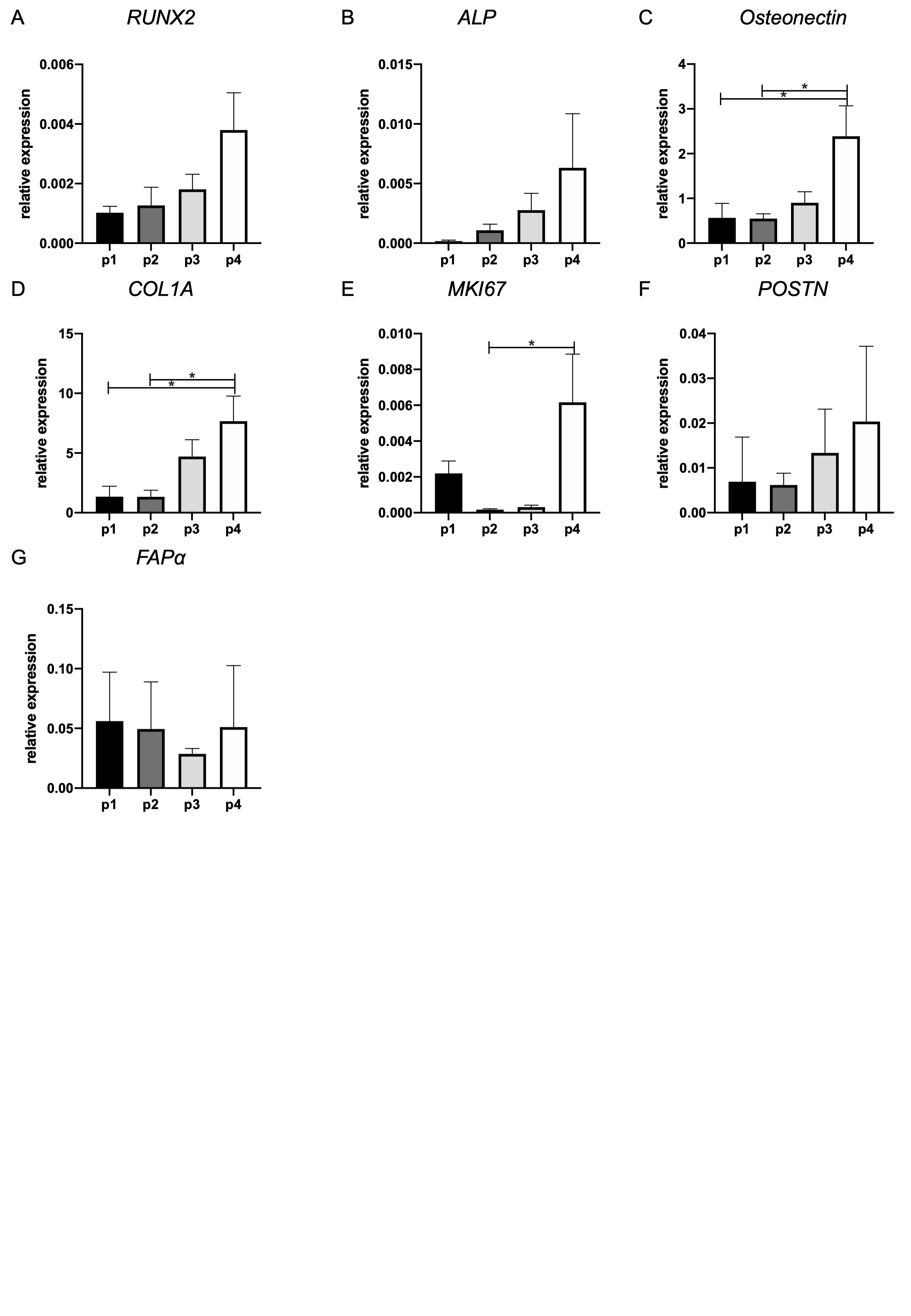

Supplement: Supplementary file 6 — Figure S6. Passaging increases the expression of the osteogenesis-related genes of gingival fibroblast cells deriving from diseased sites. Gene expression of (A) RUNX2, (B) ALP, (C) Osteonectin, (D) COL1A and (E) MKI67, (F) POSTN, and (G) FAPα. n=4. Significant results are shown (black bars). *p < 0.05 [file 13577_2023_995_MOESM6_ESM.tiff]

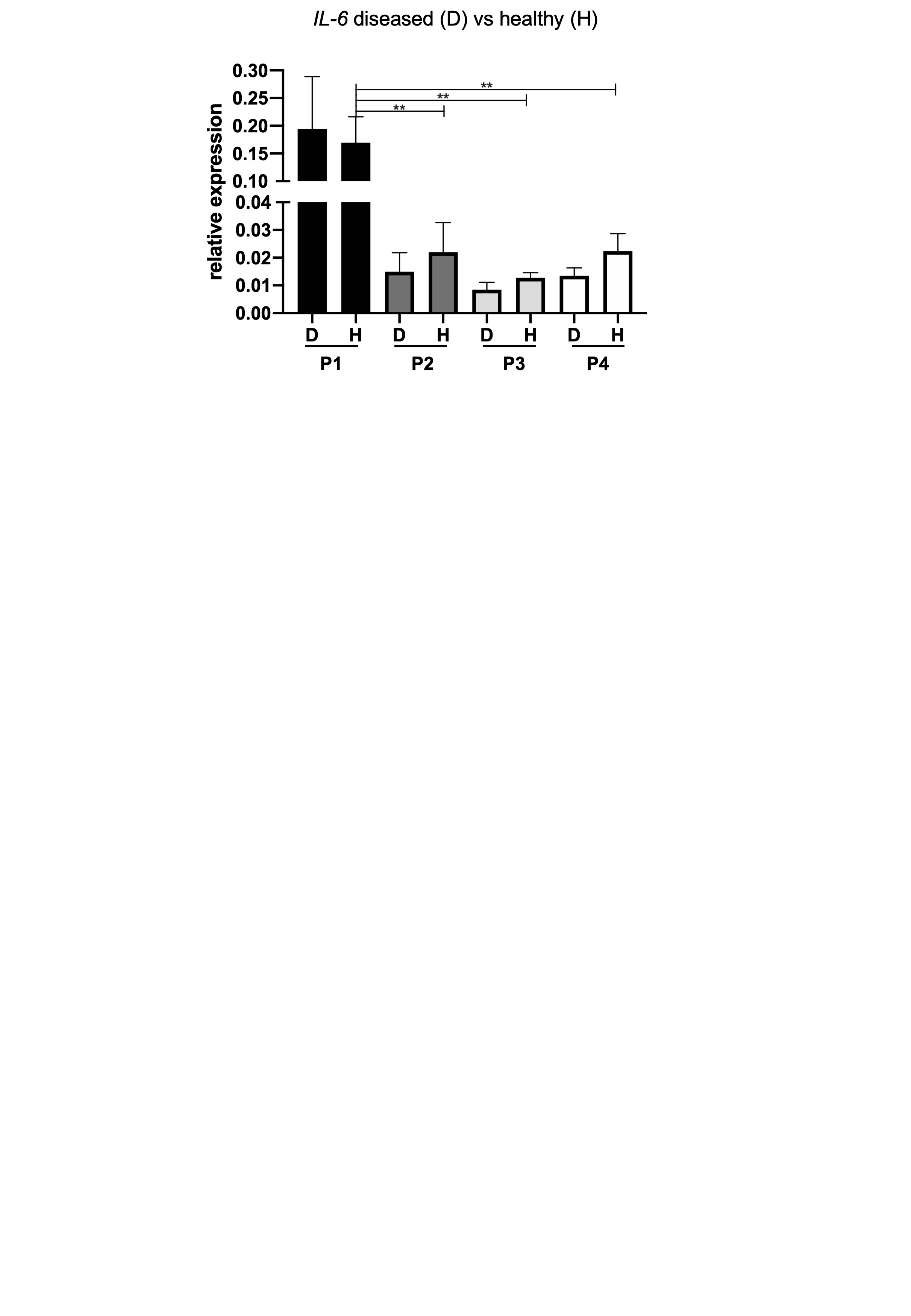

Supplement: Supplementary file 7 — Figure S7. Gene expression of IL-6 does not differ between gingival fibroblasts deriving from healthy and diseased sites. Gene expression of IL-6. n=5 for healthy and n=4 for diseased gingiva. Significant results are shown (black bars). **p < 0.01 [file 13577_2023_995_MOESM7_ESM.tiff]
